# Patterning the Face

**DOI:** 10.1371/journal.pbio.0020295

**Published:** 2004-07-20

Vertebrates come in a dazzling array of shapes and sizes, from blue whales to pygmy bats, their overt morphology determined largely by the skeleton. The head skeleton in particular has undergone remarkable diversification, as is beautifully illustrated in Darwin's examination of beak morphology in Galapagos finches. It is now appreciated that a large part of the facial skeleton is derived from a newly identified, vertebrate-specific population of cells, called the cranial neural crest, that has its origins at the border of the dorsal neural plate (the future brain).

Vertebrates develop from three germ layers—the endoderm, mesoderm, and ectoderm—which each give rise to distinct elements in the emerging body plan, and interactions between these layers are a common feature of embryogenesis. For example, early in development, cranial neural crest cells migrate to positions along the bottom (ventral side) of the future head, where they form a series of developmental intermediate structures called pharyngeal arches. The arches facilitate interactions between crest cells (derived from ectoderm) and neighboring tissues (such as endoderm and surface ectoderm), which induce specific bone and cartilage patterns in the face. Recent chick studies showed that head endoderm, which contributes to the lining of the pharynx and gills, can pattern the facial skeleton. But the question remained, by what mechanism does endodermal signaling induce specific patterns of cartilage and bone?

In this issue of *PLoS Biology*, Justin Crump, Mary Swartz, and Charles Kimmel study the patterning of a jaw-support cartilage called the hyosymplectic in the larval zebrafish and find a “hierarchy of tissue interactions” at work. In zebrafish mutated for a gene called *integrin*α*5*, the authors report, a specific region of the hyosymplectic cartilage fails to develop. The loss of this cartilage region correlates with the loss of the first endodermal pouch. Pouches are outpocketings of the head endoderm that fuse with the skin to form the gill slits later in development. By labeling individual crest cells with fluorescent dye and making time-lapse recordings of these cells in transgenic fish, Crump et al. show that the hyosymplectic cartilage regions lost in the *integrin*α*5* mutant are normally derived from crest cells directly adjacent to the first pouch.[Fig pbio-0020295-g001]

**Figure pbio-0020295-g001:**
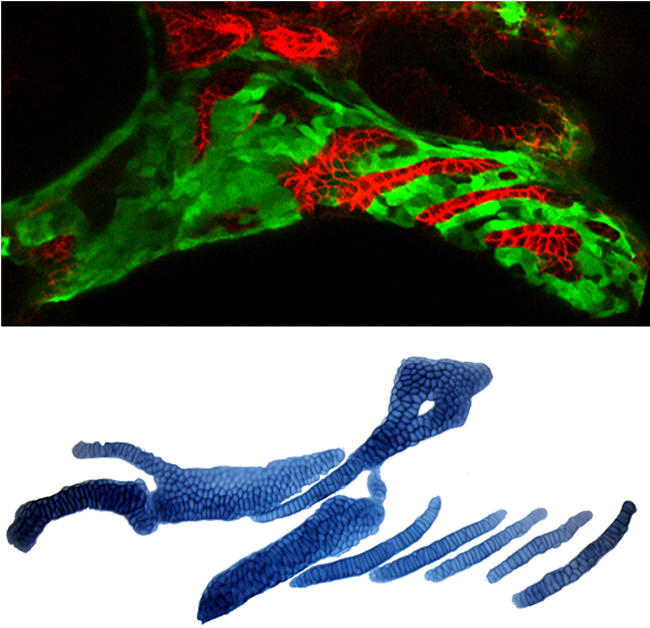
Pharyngeal development in a zebrafish embryo

Integrins are transmembrane receptors that promote cell adhesion and signaling. Although integrins function in crest cell migration, Crump et al. show that the Integrinα5 receptor is required in endoderm for hyosymplectic cartilage development and appears to promote development of the first pouch. The first pouch in turn acts as a template, by promoting both the survival and local clustering of crest cells, to pattern a specific region of the hyosymplectic cartilage.

But the pouch may have more far-reaching effects. Since *integrin*α*5* mutants also have region-specific defects in cranial muscles and nerves, the first pouch may serve to organize an entire functional unit in a region of the head. As the hyosymplectic element has undergone considerable change during evolution—from a jaw-support element in fish to a tiny, sound-conducting bone called the stapes in mammals—Crump et al. speculate that such a local, interconnected strategy of development would facilitate evolution of the vertebrate head. Changes in endodermal signaling would allow a particular skeletal element to vary in shape or size, in coordination with the muscles and nerves that move the skeletal element and independent of other regions of the head.

It will be interesting to determine, the authors note, whether this hierarchical organization applies to other skeletal elements in the head. But for now, these results will inform efforts to understand the specificity of interrelated defects seen in human craniofacial syndromes such as DiGeorge Syndrome, whose underlying causes lie in the development of the endoderm.

